# Assessment of lower incisor position and symphysis dimensions among different skeletal patterns in the Chhattisgarh population

**DOI:** 10.6026/973206300222653

**Published:** 2026-04-30

**Authors:** E Reshma, Virendra Vadher, Chhaya Barapatre, Shalabh Baxi, Shweta Singh, Gangesh B Singh

**Affiliations:** 1 Department of Orthodontics and Dentofacial Orthopedics, Government Dental College, Raipur, Chhattisgarh, India

**Keywords:** Lower incisors, mandibular symphysis, skeletal malocclusion, cephalometrics

## Abstract

Lower incisor position and mandibular symphysis morphology across different sagittal skeletal malocclusions, which complicates orthodontic diagnosis and treatment planning is of interest. Therefore, it is of interest to evaluate these parameters among skeletal Class I, Class II and Class III individuals. A cross-sectional analysis was conducted on 180 untreated subjects aged 16-45 years using standardized lateral cephalometric radiographs, with groups classified based on the ANB angle. Statistical analysis using one-way ANOVA and Tukey's post-hoc test identified significant intergroup differences. Thus, proclined lower incisors in Class II subjects and retroclined incisors with increased symphyseal dimensions in Class III subjects, highlighting important dentoalveolar and skeletal variations.

## Background:

The dentofacial complex possesses an inherent compensatory mechanism that functions to maintain facial harmony and proportionality. Dental compensation aims to establish a functional incisor relationship by masking underlying anteroposterior and vertical skeletal discrepancies. In Class III malocclusion, the lower incisors are typically retroclined. In contrast, the maxillary incisors are proclined, whereas in the vertical dimension, incisor eruption and variations in symphyseal length contribute to dentoalveolar adaptation [1, 2]. The position of the mandibular incisors is considered a key element in orthodontic diagnosis and treatment planning, as it plays a critical role in maintaining occlusal stability and facial balance. The close relationship between form and function, as explained by evolutionary principles, is evident in orthodontics through skeletal and more prominently, dentoalveolar compensations, whereby the dentition adapts to inherent skeletal imbalances [3]. Angle's orthodontic philosophy emphasized the achievement of ideal occlusion as a stimulus for normal maxillomandibular development and facial balance, placing relatively little importance on mandibular incisor inclination or tooth-jaw size discrepancies in early orthodontic concepts. However, subsequent studies have demonstrated that mandibular incisors play a predominant role in dentoalveolar compensation, often adapting their inclination to maintain occlusal harmony in the presence of skeletal disharmony. During orthodontic treatment, teeth tend to move toward a new equilibrium, underscoring the importance of respecting biological limits when planning incisor movement [4, 11 and 12]. The mandibular symphysis is a critical anatomical structure influencing facial profile esthetics. It serves as a determining factor in decisions regarding the positioning of lower incisors in both orthodontic camouflage and orthognathic surgical cases [5, 7]. Its morphology is affected by anteroposterior skeletal relationships, with variations in symphyseal width and height influencing the extent of permissible orthodontic tooth movement [6, 8 and 10]. A wider symphysis may allow greater proclination of the lower incisors, whereas a narrow symphysis may restrict dentoalveolar compensation and increase the risk of alveolar bone dehiscence [9, 11]. Additionally, functional factors such as masticatory forces, vertical skeletal pattern, mandibular length and lower incisor inclination contribute to adaptive changes in symphyseal morphology. During growth, dentoalveolar compensation associated with sagittal discrepancies may further modify symphyseal dimensions, while vertical growth direction can indirectly influence mandibular position and symphyseal form [8, 9, 10 and 13]. Therefore, it is of interest to understand these relationships, which are essential for establishing safe and effective orthodontic treatment goals within the biological limits of the mandibular symphysis.

## Materials and Methods:

This cross-sectional cephalometric study was conducted to assess the position of the lower incisors and the dimensions of the mandibular symphysis in subjects with different skeletal patterns from the Chhattisgarh population. The study sample consisted of pretreatment lateral cephalograms of individuals who reported to the Department of Orthodontics and Dentofacial Orthopedics of a dental teaching institution in Chhattisgarh. Ethical clearance for the study was obtained from the Institutional Ethics Committee (IEC Proposal No. 7661/GDC/Institutional Ethics Committee/2023) and all procedures were performed in accordance with ethical standards. A total of 180 subjects aged 16-45 years were included. The inclusion criteria comprised individuals with permanent dentition, no prior history of orthodontic treatment, good- quality lateral cephalograms and no craniofacial anomalies, trauma or systemic conditions affecting craniofacial growth. Subjects with missing teeth, gross facial asymmetry, or poor radiographic records; patients who had undergone prior orthodontic treatment; and patients with root resorption were excluded. The skeletal pattern was determined using the ANB angle and subjects were categorized into skeletal Class I, Class II and Class III groups. Standardized lateral cephalograms were obtained with the teeth in centric occlusion and lips in a relaxed position. All radiographs were traced manually by a single investigator to minimize inter-observer variability. Acetate tracing paper of 0.003-inch matte finish and 0.3mm HB lead pencil was used for tracing of the lateral cephalogram. Lower incisor position was assessed using angular and linear measurements relative to mandibular planes, including incisor mandibular plane angle and linear distance from the incisor apex to the mandibular plane. Mandibular symphysis dimensions were evaluated by measuring symphysis height, width and thickness at defined anatomical reference points. All landmarks were identified according to standardized cephalometric definitions.

## Statistical analysis:

The collected data were tabulated and analyzed using SPSS software. Descriptive statistics, including the mean and standard deviation, were calculated. Intergroup comparisons were performed using one-way ANOVA followed by post-hoc tests. The level of statistical significance was set at p < 0.05.

## Results:

Cephalometric evaluation was conducted on 180 subjects, divided equally into skeletal Class I, Class II and Class III malocclusions. The results include comparative analysis using Welch's One-Way ANOVA and Tukey's HSD post-hoc tests. The aim was to identify significant skeletal, dental and symphyseal variations among the three groups and determine the parameters most strongly associated with each malocclusion type. Table 1 (see PDF) shows the frequency of the parameters used in the study in terms of mean ± SD in Class I, Class II, Class III malocclusion and skeletal variables with significant differences included: GO-GN to SN (p = 0.002), ID-Me (p = 0.017), Id-B (p < 0.001), ID-B-POG (p = 0.003), AIPHA (p < 0.001), B-B1 Gn (p = 0.013). Figure 1 (see PDF) shows that IMPA was larger in the Class II group (103 ± 8.997) than in Class I and Class III (Class II> Class I> Class III). FMIA was larger in the Class III group (70.017 ± 8.985) than in Class I and Class II (Class III > Class I > Class II). L1-NB, L1-A POG and L1-N POG were larger in the Class II group as compared to the other two groups. ID-B, ID ME and ALPHA were larger in the Class III group as compared to the other two groups. These findings indicate that mandibular plane angle, lower facial height, chin prominence and symphyseal morphology differ significantly across malocclusion classifications. The strong significance seen in AIPHA and Id-B underscores the intimate relationship between skeletal pattern and symphyseal anatomy. Dental variables showed the most robust statistical differences. The following parameters exhibited highly significant results (p<0.001): MI-OP, IMPA, FMIA, L1-NB (linear, angular) and Interincisal Angle. These results confirm that underlying skeletal discrepancies strongly influence dental compensations in lower incisors. Class II subjects consistently demonstrated protrusive and proclined incisors, while Class III individuals showed retroclination and increased interincisal angle, reflecting their compensatory mechanism. The mandibular plane angle (GO-GN to SN) differed significantly between groups. Class II subjects displayed the highest mean angle (30.567°C ± 7.439), indicating a steeper mandibular plane, followed by Class I (27.650°C ± 6.202) and Class III (25.983°C±6.353). This gradient reflects the more hyperdivergent skeletal pattern often associated with Class II profiles. Anterior facial height components also showed variation. ID-Me (Total length of MS), representing inferior facial height, was greatest in Class III (26.600 ± 3.300) and smallest in Class II (24.817±3.938), suggesting shorter lower facial dimensions in Class II individuals. Similarly, Id-B (Figure 2 - see PDF), another symphyseal depth indicator, was highest in Class III (7.517 ± 3.281) and markedly lower in Class II (4.900±1.773), demonstrating increased chin prominence in Class III. L1-NB, L1-A Pog, L1-N Pog and IMPA-showed noticeable trends. Class II subjects had the most proclined incisors, as reflected by higher values of L1-NB (8.833±3.542 mm) and IMPA (103°C ± 8.997°C). In contrast, Class III subjects exhibited the greatest retroclination, with L1-NB linear as low as 5.083 ± 2.657, L1-NB angular 24.583 ± 7.345 and IMPA at 90.25°C ± 16.430. FMIA demonstrated an opposite relationship, being highest in Class III (70.017°C ± 8.985) and lowest in Class II (52.667°C ± 8.386), again illustrating compensatory retroclination in Class III individuals. The Interincisal Angle differed between groups, being highest in Class III (119.817°C ± 11.651) and lower in Class I and II (110.417°C±18.922 and 110.467°C ± 15.047, respectively). This reflects the upright or retroclined incisor posture commonly observed in skeletal Class III cases. Notably, more morphologically significant values such as AIPHA, B-B1-Gn and ID-B POG exhibited pronounced differences. AIPHA was highest in Class III (103.667°C±8.870) and lowest in Class II (92.367°C ± 9.135), denoting increased alveolar housing angulation in Class III. B-B1-Gn and ID-B-POG are higher in class III.

## Discussion:

The present study evaluated lower incisor position and mandibular symphysis dimensions across different sagittal skeletal patterns. The findings demonstrated statistically significant variations in both dental and skeletal parameters among Class I, Class II and Class III subjects, highlighting the close relationship between lower incisor inclination, symphyseal morphology and the underlying craniofacial pattern. These observations are consistent with previous studies that emphasize the role of dentoalveolar compensation in maintaining functional occlusion in the presence of skeletal discrepancies. In the sagittal skeletal groups, Class II subjects exhibited the most proclined lower incisors, as evidenced by higher L1-NB and IMPA values and a reduced FMIA. This proclination represents a compensatory mechanism to camouflage mandibular retrusion, a finding supported by Nazir and Mushtaq and by Farrukh *et al.* who reported similar trends in Class II malocclusions [14, 20]. Conversely, Class III subjects demonstrated retroclined lower incisors, increased frankfort-mandibular incisor angle (FMIA) and interincisal angles, reflecting compensation for a prognathic mandible. Class I subjects showed intermediate values, corresponding to their relatively balanced skeletal pattern. These findings align with reports by Zhang *et al.* and Aristide *et al.* who documented more upright lower incisors in Class III individuals [18, 21]. Skeletal and chin-related parameters also varied significantly among the groups. The mandibular plane angle was highest in Class II subjects, confirming their tendency toward a hyperdivergent growth pattern. In contrast, Class III subjects exhibited greater chin prominence, as indicated by increased Id-B, Id-Me, Id-B-Pog and ID-B-POG values. Similar observations have been reported by Saeed *et al.* and Hernández-Sayago *et al.* who noted increased symphyseal height and chin prominence in Class III skeletal patterns [15, 16]. Class II subjects demonstrated smaller symphyseal dimensions, consistent with findings from Gomez *et al.* and Sharma *et al.* [17, 22]. Despite differences in absolute symphyseal measurements, proportional ratios remained relatively constant across skeletal classes, suggesting that overall symphyseal shape is preserved regardless of sagittal pattern. Earlier studies building on Steiner's analysis proposed a multiple-regression model that incorporates ANB and other skeletal parameters to predict lower incisor position and also a further significant association between ANB angle and lower incisor inclination, supporting the influence of maxillomandibular relationships on incisor positioning was established [23]. However, due to limitations in ANB angle variability, using the Wits appraisal is considered as a more stable sagittal indicator. Despite these limitations, the present study employed the ANB angle for skeletal classification, consistent with several earlier investigations [14, 20]. The close relationship between lower incisor inclination and the mandibular plane angle, a concept strongly supported by the present findings was emphasized in this study. A statistically significant association was observed between lower incisor inclination and mandibular plane angle across all skeletal groups, reinforcing the influence of vertical growth patterns on dentoalveolar compensation [16]. Cephalometric radiography remains an essential diagnostic tool in orthodontic treatment planning, particularly in decisions involving extraction versus non-extraction therapy. While Angle initially emphasized occlusal interdigitation, Tweed later highlighted the importance of maintaining the lower incisors within the basal bone for long-term stability. Tweed suggested that an IMPA of approximately 90°C, within the 85-95°C range, represents an optimal balance between function and facial esthetics. In the present study, Class II subjects demonstrated higher mean IMPA values. In comparison, Class III subjects showed significantly lower values, indicating more upright incisors in prognathic mandibles and more proclined incisors in retrognathic mandibles. This pattern mirrors findings reported by Nazir and Mushtaq, Farrukh *et al.* and Zhang *et al.* [14, 20 and 21]. The morphology of the mandibular symphysis plays a crucial role in determining the safe limits of orthodontic tooth movement. Saeed *et al.* emphasized that the surrounding alveolar bone constrains the inclination and position of lower incisors and excessive movement beyond these limits may result in periodontal compromise [15]. Gomez *et al.* reported that symphyseal height was significantly reduced in hyperdivergent Class II individuals, a finding corroborated by the present study, which demonstrated significantly smaller B-B1-Gn measurements in Class II subjects compared with Class I and Class III groups [17]. Chin concavity, represented by the Id-B-Pg angle, was more pronounced in Class III subjects in the present study. These findings are consistent with reports by Yetgin *et al.* and Aristide *et al.* who observed reduced anterior symphyseal concavity in Class III patients due to compensatory retroclination of the lower incisors [18, 19]. This retroclination induces remodeling of the alveolar bone, resulting in a flatter anterior symphyseal contour. Similar adaptive changes have been described by Saeed *et al.* and Gomez *et al.* reinforcing the concept of skeletal-dental interdependence [15, 17]. Cephalometric has been adapted as an important clinical tool for assessment of jaw relationship in all three planes-anteroposterior, transverse and vertical being an integral part of orthodontic treatment planning after its discovery. Despite its strengths, the present study has certain limitations. The relatively small sample size may limit generalizability and the use of two-dimensional lateral cephalograms restricts assessment to linear and angular measurements, which may not fully represent true three-dimensional symphyseal morphology. Future studies incorporating larger samples and CBCT-based analyses are recommended to provide more comprehensive insights into the relationship between lower incisor position and mandibular symphysis morphology [19]. The findings of this study emphasize the importance of evaluating the position of the lower incisors in relation to mandibular symphysis morphology in orthodontic diagnosis and treatment planning. Understanding these relationships is essential for determining safe limits of tooth movement, preventing periodontal damage and achieving stable and esthetically pleasing treatment outcomes, particularly in borderline extraction and camouflage cases.

## Conclusion:

Cephalometric evaluation of the selected sample provided a comprehensive understanding of how dental and skeletal components interact to achieve functional and esthetic balance in the presence of underlying skeletal discrepancies. Data shows that both lower incisor position and symphyseal morphology are significantly influenced by sagittal skeletal pattern.

## Declarations:

## Author contribution:

All authors contributed extensively to the study. Reshma E. conceptualised the research, designed the technique and led data collection. Chhaya Barapatre supervised the examination and selection of subjects, tested the method and executed statistical analysis. Shalabh Baxi performed the literature assessment, interpreted records and contributed to manuscript writing. Reshma E. and Shweta Singh were responsible for collecting clinical data. Gangesh B. Singh handled record analysis, organized figures and tables and ensured adherence to ethical recommendations. Virendra Vadher conducted the final assessment, edited the manuscript and facilitated investment acquisition. All authors reviewed and accepted the very last version of the manuscript.

## Funding:

None

## Institutional review board statement:

Ethical approval was acquired before initiating the study with IEC Proposal No. 7661/GDC/Institutional Ethics Committee/2023.
